# Assessing the effects of distinct biologic therapies on rheumatoid arthritis pain by nociceptive, neuropathic and nociplastic pain components: a randomised feasibility study

**DOI:** 10.1186/s40814-024-01505-4

**Published:** 2024-05-16

**Authors:** Liban Ahmed, Kathryn Biddle, Anna Blundell, Soraya Koushesh, Patrick Kiely, Gill Mein, Philip Sedgwick, Nidhi Sofat

**Affiliations:** 1 Institute for Infection and Immunity, St George’s University of London, Cranmer Terrace, London, SW17 ORE UK; 2 Department of Rheumatology, St George’s University Hospitals NHS Foundation Trust, London, UK; 3 Institute of Medical and Biomedical Education, St George’s, University of London, Cranmer Terrace, London, SW17 ORE UK; 4 Centre for Allied Health, St George’s, University of London, Cranmer Terrace, London, SW17 ORE UK

**Keywords:** Rheumatoid arthritis, Pain, Biologics, Adalimumab, Abatacept, Nociceptive, Neuropathic, Nociplastic pain, Stratification

## Abstract

**Background:**

Pain management is a major unmet need in people with rheumatoid arthritis (RA). Although many patients are treated with disease modifying anti-rheumatic drugs (DMARDS), including biologic therapies, many people with RA continue to experience significant pain. We aimed to determine whether performing a comprehensive pain evaluation is feasible in people with active RA receiving conventional DMARDs and biologic therapies.

**Methods:**

The BIORA-PAIN feasibility study was an open-label, randomised trial, which recruited participants suitable for treatment with biologic therapy. The primary feasibility outcomes were recruitment, randomisation and retention of eligible participants. All participants underwent pain assessment for nociceptive, neuropathic and nociplastic pain during the 12-month study period, with quarterly assessments for VAS (Visual Analogue Scale) pain, painDETECT and QST (quantitative sensory testing). This trial was registered in clinicaltrials.gov NCT04255134.

**Results:**

During the study period, 93 participants were screened of whom 25 were eligible: 13 were randomised to adalimumab and 12 to abatacept. Participant recruitment was lower than expected due to the COVID-19 pandemic. Pain assessments were practical in the clinical trial setting. An improvement was observed for VAS pain from baseline over 12 months, with a mean (SEM) of 3.7 (0.82) in the abatacept group and 2.3 (1.1) in the adalimumab group. There was a reduction in painDETECT and improvement in QST measures in both treatment groups during the study. Participant feedback included that some of the questionnaire-based pain assessments were lengthy and overlapped in their content. Adverse events were similar in both groups. There was one death due to COVID-19.

**Conclusions:**

This first-ever feasibility study of a randomised controlled trial assessing distinct modalities of pain in RA met its progression criteria. This study demonstrates that it is feasible to recruit and assess participants with active RA for specific modalities of pain, including nociceptive, neuropathic and nociplastic elements. Our data suggests that it is possible to stratify people for RA based on pain features. The differences in pain outcomes between abatacept and adalimumab treated groups warrant further investigation.

**Trial registration:**

NCT04255134, Registered on Feb 5, 2020.

## Key messages

What uncertainties existed regarding the feasibility?

Prior to the study, it was uncertain whether it is feasible to conduct a panel of pain assessments using quantitative sensory testing (QST) and patient reported outcome measures (PROMs) in a clinical setting.

What are the key feasibility findings?

Our study found that it is feasible to combine quantitative sensory testing (QST) with pain questionnaires to assess RA pain at 3 monthly intervals over 12 months during the study period. However, there was some overlap between questions in the PROMSs used. There were no adverse effects found related to pain testing.

What are the implications of the feasibility findings for the design of the main study?

It is feasible to combine QST with PROMS for a comprehensive pain assessment in people with RA in a clinical setting. The number of questionnaires administered could be reduced in the main study to minimise repeatability of questions covered by the range of PROM questionnaires used.

## Introduction

Rheumatoid arthritis (RA) is a common condition, affecting 1–2% of the population worldwide. With the advent of synthetic and biologic disease modifying anti-rheumatic drugs (DMARDs) for the treatment of active RA, clinicians managing patients with RA have a wide range of treatments available to control their active disease [1]. A challenge for RA management in the clinic is to stratify patients for the most appropriate treatment at the correct time to achieve optimal disease control and to maintain the patient in remission for as long as possible [2]. In the clinic, many disease modifying anti-rheumatic drugs (DMARDs) are effective in achieving suppression of inflammation, but pain remains a key issue in RA, and in some patients treated with DMARDs, pain control remains a significant unaddressed issue [3, 4].

Several distinct components of the adaptive immune system are implicated in the development and persistence of autoimmunity in RA. It is well recognised that tumour necrosis factor (TNF) alpha represents a key cytokine in mediating the inflammatory response in RA and leads to activation of numerous downstream pathways implicated in pain and inflammation [5]. It is therefore apparent how suppression of TNF alpha through biologic TNF inhibitor agents often results in suppression and control of inflammation and pain in people with RA. In contrast, T cells also play a pivotal role in RA and are implicated in the loss of tolerance and development of autoimmunity. In addition, an increasing number of studies are providing evidence for the role of T cells in mediating pain. T cells infiltrate injured sciatic nerves [6] and nerve receptors such as P2RX7 influence memory T cell function [7]. T cell deficient mice also show increased pain sensitivity [8]. Abatacept is a fusion protein composed of the Fc region of the immunoglobulin IgG1, fused to the extracellular domain of CTLA-4. By binding to CD80 and CD86, abatacept prevents the second co-stimulatory signal, required in addition to MHC combination with antigen, resulting in T cell activation. It is therefore interesting to consider how T cell modulation via abatacept influences pain in RA [9].

Schiff et al. [9] showed that in a head-to-head trial of subcutaneous abatacept, there was similar efficacious outcome based on clinical, functional and radiographic scores compared with the TNF inhibitor adalimumab. Interestingly, in a randomised, controlled, clinical trial setting, there was an improved pain outcome profile for abatacept compared with the TNF inhibitor adalimumab [9].

To our knowledge, no randomised trials have been performed to test the feasibility of assessing distinct pain modalities in RA in people receiving biologic therapy. We hypothesised that participants who are assessed by distinct modalities of pain could be stratified for specific therapies based on their pain characteristics. Our research group has developed techniques to measure pain in rheumatoid arthritis and osteoarthritis [10, 11]. These include applying the painDETECT questionnaire and demonstrating its validity in a real-world population of people with RA [10]. We aimed to conduct a feasibility study to investigate the ability to perform a comprehensive pain evaluation in participants with active RA receiving distinct biologic therapies for nociceptive, neuropathic and nociplastic pain features. We aimed to assess a range of pain modalities using the VAS (visual analogue scale) (nociceptive pain), painDETECT (neuropathic pain) questionnaires and assessment of pain sensitisation using quantitative sensory testing (QST) (nociplastic) by pain pressure algometry (PPT). This trial, if feasible, could form the exemplar for larger trials using pain stratification for RA to guide treatments.

## Methods

The BIORA-PAIN study was a feasibility randomised clinical trial in people with RA requiring treatment with biologic therapies. We tested the pain outcome profile in people with RA on treatment with abatacept or adalimumab respectively in participants attending the Rheumatology clinic at St George’s University Hospitals NHS Foundation Trust at the time of initiation of biologic therapy and for 12 months thereafter.

### Participants

The participants in our database who were receiving care for their rheumatoid arthritis with biologic treatments were screened for enrolment. Participants were identified from rheumatology clinics in Southwest London based at St Georges University Hospitals NHS Trust. Inclusion criteria were participants with active RA with a DAS28 >5.1. Participants needed to have already received conventional DMARD therapy for a period of 4 weeks prior to study drug initiation and not have achieved remission with previous treatment of conventional cDMARD therapy as per UK NICE guidelines. The age range for inclusion was 18–75 years. Exclusion criteria included pregnancy or planned pregnancy in the next 12 months, current or previous unsuccessful use of the biologics adalimumab or abatacept and co-existing other autoimmune conditions, for example, systemic lupus erythematosus, primary/secondary Sjogren’s syndrome, connective tissue disease, fibromyalgia, primary osteoarthritis and gout. Full exclusion criteria were followed as per study protocol (clinicaltrials.gov reference number was NCT04255134).

The study started in September 2020, and the last patient visit occurred on 25^th^ October 2022. Ethical approval was given by East of England Cambridgeshire and Hertfordshire Research Ethics Committee, approval number 9/EE/0382.

### Procedures

We compared the differences in pain measures in the 2 groups and evaluated the modalities of pain outcomes in our cross-sectional population using neuropathic pain assessed by painDETECT [10], nociplastic pain sensitisation evaluated by quantitative sensory testing (QST) [11] and inflammatory pain measured by the visual analogue scale (VAS) [12] questionnaires. Objective pain score comparisons using quantitative sensory testing (QST) were also performed in the abatacept and TNF inhibitor group. QST assessments were performed as previously described with a Somedic hand held algometer [11].

At baseline, pain assessment scores inclusive of VAS, painDETECT scores and DAS-28 were taken. Body mass index (BMI) was recorded, with screening blood tests before starting biologics taken inclusive of liver function tests, renal function tests, TB, hepatitis B/C testing and inflammatory markers including ESR and CRP. As part of the biologic screening, each participant required a chest radiograph to exclude infection. After baseline measures were obtained, participants were seen a further four times at 3-month intervals. Information collected at each visit included pain and function questionnaires, patient-related outcome measures (PROMs) (see outcomes section), QST, CCP (anti-Cyclic citrullinated protein antibody) status, blood and urine for inflammatory markers and biomarker testing and concomitant medications.

### Randomisation and masking

The web-based randomisation programme developed by King’s Clinical Trials Unit was used to assign participants randomly in a 1:1 ratio. The study was not blinded; this was to allow participants to receive their usual treatment more easily and to identify those who would need further treatment swiftly switched back to usual care at the end of the study. It also allowed for easier monitoring of possible adverse effects.

### Outcomes

The primary aim was to assess the feasibility of undertaking a statistically powered, randomised, controlled trial. The primary outcomes to determine feasibility were recruitment, randomisation rates and retention. We identified suitable pain measures and ascertained if they could be collected during the study. Clinical pain measures included patient reported scores, painDETECT and visual analogue scale (VAS) and objective measures of pain using PPT after 12 months of treatment. The VAS outcome measure is a validated measure for inflammatory pain and forms part of the IMMPACT guidelines in clinical studies [12]. The painDETECT questionnaire has been increasingly adopted to assess participants for inflammatory and neuropathic pain elements in RA [10], with a measure of 0–12 suggestive of inflammatory pain, 13–18 suggesting possible neuropathic elements and 19–38 suggesting a likely neuropathic component. Participants were also assessed for nociplastic pain components using quantitative sensory testing (QST), as described above. Subjects were also stratified by CCP (anti-cyclic citrullinated peptide antibody) status. Additional clinical characteristics were obtained, including the inflammatory markers C-reactive protein (CRP) and ESR (erythrocyte sedimentation rate) plus the Hospital Anxiety and Depression Scale (HADS) [13], Health Assessment Questionnaire Disability Index (HAQ DI) [14], SF36 (36-item short form survey) [15] and PROMIS-29 (which measures physical function, fatigue, pain interference, depressive symptoms, anxiety, ability to participate in social roles and activities and sleep disturbance) [16].

A brief description of the questionnaires and their rationale for use is discussed as follows: the Health Assessment Questionnaire-Disability Index (HAQ-DI) is the most widely used measure of function in inflammatory arthritis [14]. Worse HAQ scores are associated with an increased risk of all-cause mortality. The SF36 (Short Form 36 survey) is a patient reported outcome for quality of life measures, including limitations in physical activities due to health issues, bodily pain, mental health and fatigue [15]. The PROMIS-29 is a 4-item form for physical function, fatigue, depression, anxiety, sleep disturbance and satisfaction with participation in social roles [16].

#### Quantitative sensory testing (QST)

Sites were selected to assess peripheral and central sensitisation, including the sternum, hand (30 joints including both hands and wrists), knees and malleoli. The pain pressure threshold (PPT), which was the value recorded when the pressure stimulus was perceived as pain, was measured (in kPa) at each site 3 times and a mean of all 3 values reported.

#### HAQ-DI (Health Assessment Questionnaire–Disability Index)

It is measured via 20 items, which are divided into 8 categories: dressing, rising, walking, reach, grip, hygiene and usual activities. Those with a high HAQ score at baseline or 1 year are associated with worse function outcomes in the long term.

#### PROMIS-29 outcome measure (Patient Reported Outcomes Information System)

Each item is ranked from 1to 5; therefore, the score can range from 4 to 20. The scores are converted to T scores, where the mean for the normal population is 50 and the standard deviation is 10. A larger number, above 50, represents a positive score and an increase in the item being measured. If the score is 60 for physical function, the participant has greater than normal physical function.

All adverse events were recorded and reported according to MHRA guidelines. An independent Data Monitoring Committee was chaired by an independent clinician (Dr Andrew Hitchings) and reviewed by the Trial Management Group and Trial Steering Committee.

### Sample size

As this was a feasibility trial, no formal sample size calculation was used, and the study was not powered to test the relative efficacy of adalimumab versus abatacept.

We initially estimated that 60 participants would need to be recruited, which would allow us to observe the variability of the efficacy of the outcomes to power a full trial. A proportionately reduced sample size, which required implementation due to recruitment challenges during the Covid pandemic, was still felt to be sufficient to test the primary feasibility outcomes, which was agreed with the Clinical trials unit and trial steering committee based on previous feasibility study sample sizes [17–22]. Following the revised sample size, 25 participants were recruited to the study.

### Statistical analysis

The analysis and presentation of results adhered to the CONSORT guidelines [17]. All analyses followed the intention-to-treat principle: All randomly allocated participants were analysed according to the group they were allocated to, irrespective of the intervention they received. Demographic and clinical data were presented as frequencies and percentages for categorical variables and mean and standard deviation for normally distributed continuous variables. Statistical hypothesis testing was not performed since this was a feasibility study.

The study planned to investigate the pain responses in subjects with RA receiving biologic therapies. The trial was not powered to test or demonstrate efficacy of one biologic over another. Outcome analyses were conducted according to the statistical analysis plan described in the protocol. Clinical primary outcomes for pain and QST were calculated as proportions with corresponding binomial exact 95% CIs. Missing data were imputed by scoring guidelines and pre-defined rules as specified for the corresponding questionnaire. Data were analysed using IBM SPSS Statistics 29 for analysis and trends in outcomes in each treatment group. Graphpad Prism 9 was used for graphical illustration.

### Role of the funding source

The funder of the study had no role in the study design, data analysis, data interpretation, writing of the report, or the decision to submit the manuscript for publication.

## Results

**Fig. 1:**
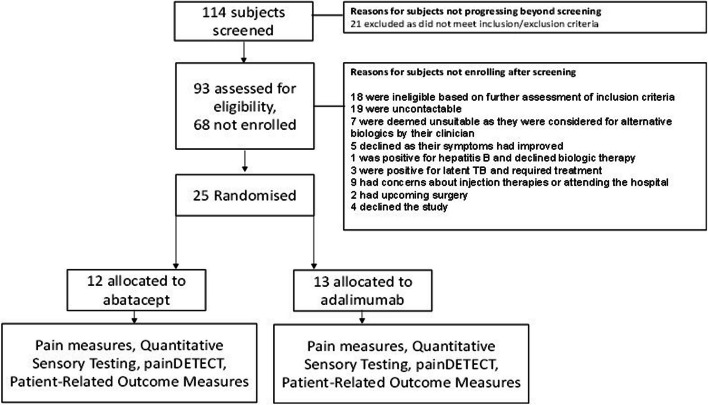
BIORA-PAIN study CONSORT Flow diagram

The BIORA-PAIN study recruited participants from September 2020 with the last patient last visit on October 2022. A total of 93 subjects were screened. There were 21 subjects excluded after screening as subjects did not meet the full inclusion and exclusion criteria. There were 93 participants assessed for eligibility, of which 25 were enrolled. The factors influencing non-enrolment are summarised in the CONSORT flow diagram (Fig. 1). The most common reasons included participants that were not contactable during the COVID-19 pandemic, being considered for alternative biologic therapy, or participants who declined to be in the trial due to concerns about new treatments during the pandemic. The remaining 25 participants were randomised to abatacept (*n* = 12) and adalimumab (*n* = 13). Of the 114 participants screened, 93 (81.6%; 95% CI 74.5%, 88.7%) were assessed for eligibility. Of the 93 patients assessed for eligibility, 25 (26.9%; 95% CI 17.9%, 35.9%) were recruited and randomised.

Due to the COVID-19 pandemic, there was a clinical study pause from December 2020 to March 2021, which reduced the study recruitment period to 14 months instead of 18 months. Furthermore, the impact on participant recruitment due to conducting the trial during the COVID-19 pandemic was that several participants were understandably reluctant to attend physically for assessment. After statistical review, it was deemed that smaller group sizes of 8–10 was still within the recommendations of the authoritative papers on groups sizes in feasibility trials [17–22].

Of the 25 participants that were randomised, six were subsequently withdrawn due to not meeting the full inclusion/exclusion criteria since they had secondary Sjogren’s syndrome. The protocol deviation was reported to the Medicines and Healthcare Products Regulatory Agency (MHRA). Of the 19 eligible participants recruited to the trial and randomised to treatment, two did not complete the 12 months’ treatment due to lack of efficacy in the trial. Furthermore, there was one death (due to COVID-19), one participant experienced a SUSAR (Suspected Unexpected Serious Adverse Reaction), whilst further two participants withdrew due to urinary tract infections (UTI). In total, 13 of the 19 (68%; 95 CI: 48% to 89%) participants that were eligible to take part in the trial achieved the progression criteria—defined as the completion of the trial for the full 12-month period.

### Feasibility of performing pain assessments during study period

In enrolled participants, there was good acceptability for attending study visits and having their pain outcomes assessed. Each visit took approximately 90–120 min in order to complete the pain questionnaires and QST. Feedback that we obtained from some of the participants was that there were a high number of questionnaires requiring completion, with some overlap of information duplication in the questionnaires. In a few cases, participants did not complete answers to a specific question in a questionnaire. If this was noticed by the study team during the study visit, the participant was asked to go back and complete the question. If it was not noticed during the study visit, then the unanswered question was handled as missing data during the analysis. There were no withdrawals due to lack of compliance or adherence to pain assessments.

### Demographics of randomised participants

Study demographics are shown in Table 1. There was no noticeable difference in mean age between the groups. Participants randomised to abatacept had a mean (SD) age of 56.5 (13.5) and for adalimumab a mean age of 53.9 (12.4). A slightly higher proportion of the abatacept group were female: 91.7 % (*n* = 11) versus 84.6 % (*n* = 11) in the adalimumab group. There were 53.8% (*n* = 7) White subjects and 46.2% (*n* = 6) Asian subjects in the adalimumab group. In the abatacept group, 83.4% (*n* = 10) were White, 8.3% (*n* = 1) Afro-Caribbean and 8.3% (*n* = 1) Asian.

**Table 1:** Baseline demographics and clinical characteristics (intention-to-treat population)

	SC abatacept + MTX (*N* = 12)	Adalimumab + MTX (*N* = 13)
**Age, years (SD)**	56.5 ±13.5	53.9 ±12.37
**Female Sex (%)**	91.7%	84.6
**Race, white (%)**	83.4%	53.8%
**Asian (%)**	8.3%	46.2%
**Afro-Caribbean**	8.3	0
**CRP -mg/dL (SD)**	12.7 (10.8)	8.31 (9.8)
**Score on DAS-28 (CRP)**^**b**^** (SD)**	5.8 (0.5)	5.8 (0.5)
**Score on HAQ-Disability Index**^**a**^** (SD)**	1.6 (0.8)	1.4 (0.6)
**Score on PainDETECT**^**c**^** (SD)**	16.4 (4.4)	17.5 (7.8)
**Score on VAS (SD)**	6.1 (1.6)	5.6 (2.2)
**Score on QST Hand§ (SD)**	251.1 (202.3)	237.4 (134.7)
**Score on QST Large Joint§ (SD)**	285.3 (193.3)	253.7 (139.9)
**Score on QST Sternum**^**d**^** (SD)**	183.6 (131.7)	167.6 (95.8)
**Positive for CCP -no. (%)**	10 (83.3%)	9 (69.2%)
**Smoker, no.**	2	2
**BMI (SD)**	29.2 (12)	30.2 (7)

^a^The degree of disability was assessed with the use of Health Quality Assessment Questionnaire (HAQ)-Disability Index, in which scores range 0–3, with higher scores indicating a greater disability

^b^Arthritis disease activity was assessed with the use of the Disease Activity Score for 28-joint counts (DAS28); scores range from 0 to 9.31, with higher scores indicating more active disease

^c^The degree of neuropathic pain was assessed with the use of painDETECT questionnaire, in which scores range from 0 to 28, with higher scores indicating a higher likelihood of neuropathic pain

^d^The degree of pain sensitisation was measured by quantitative sensory testing (QST), with higher scores indicating a better control of pain

The abatacept group had a greater mean baseline C-reactive protein (CRP)(SD) 12.7(10.8) versus 8.3 (9.8) with adalimumab. The groups were equally matched for disease activity score (DAS28); the mean DAS-28 (CRP) in the abatacept group was 5.8 (SD 0.5) and 5.8 (0.5) in the adalimumab group. The mean visual analogue score (VAS) for pain was 6.1 (SD 1.6) in the abatacept group and 5.6 (2.2) in the adalimumab group. The mean baseline painDETECT score was 16.4 (SD 4.4) in the abatacept group and 17.5 (7.8) in the adalimumab group. There were 75% (*n* = 12) participants who were CCP positive in the abatacept group, and 76.9% (*n* = 13) were positive in the adalimumab group.

### Pain assessments conducted during study

**Fig. 2:**
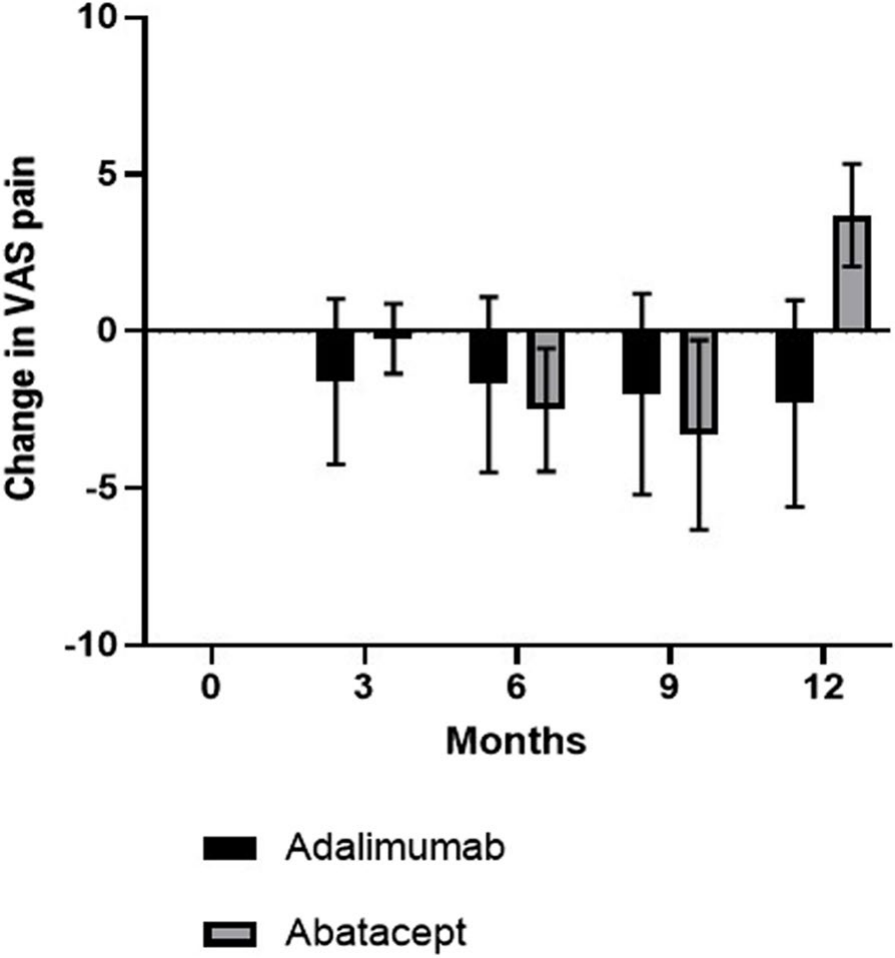
Graph illustrating change in visual analogue scale (VAS) in the adalimumab and abatacept groups over 12 months’ treatment. Box and whisker plots show mean and standard deviations

**Fig. 3:**
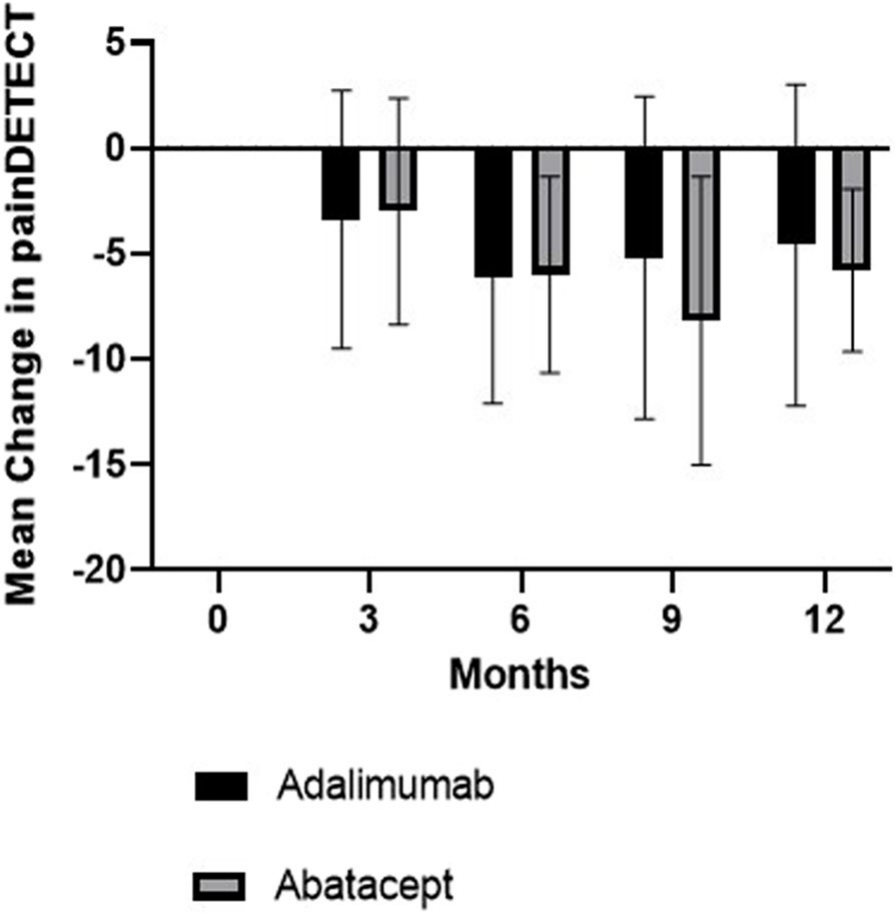
Graph illustrating change in painDETECT in adalimumab and abatacept groups respectively over 12 months’ treatment. Box and whisker plots show mean and standard deviations

**Fig. 4:**
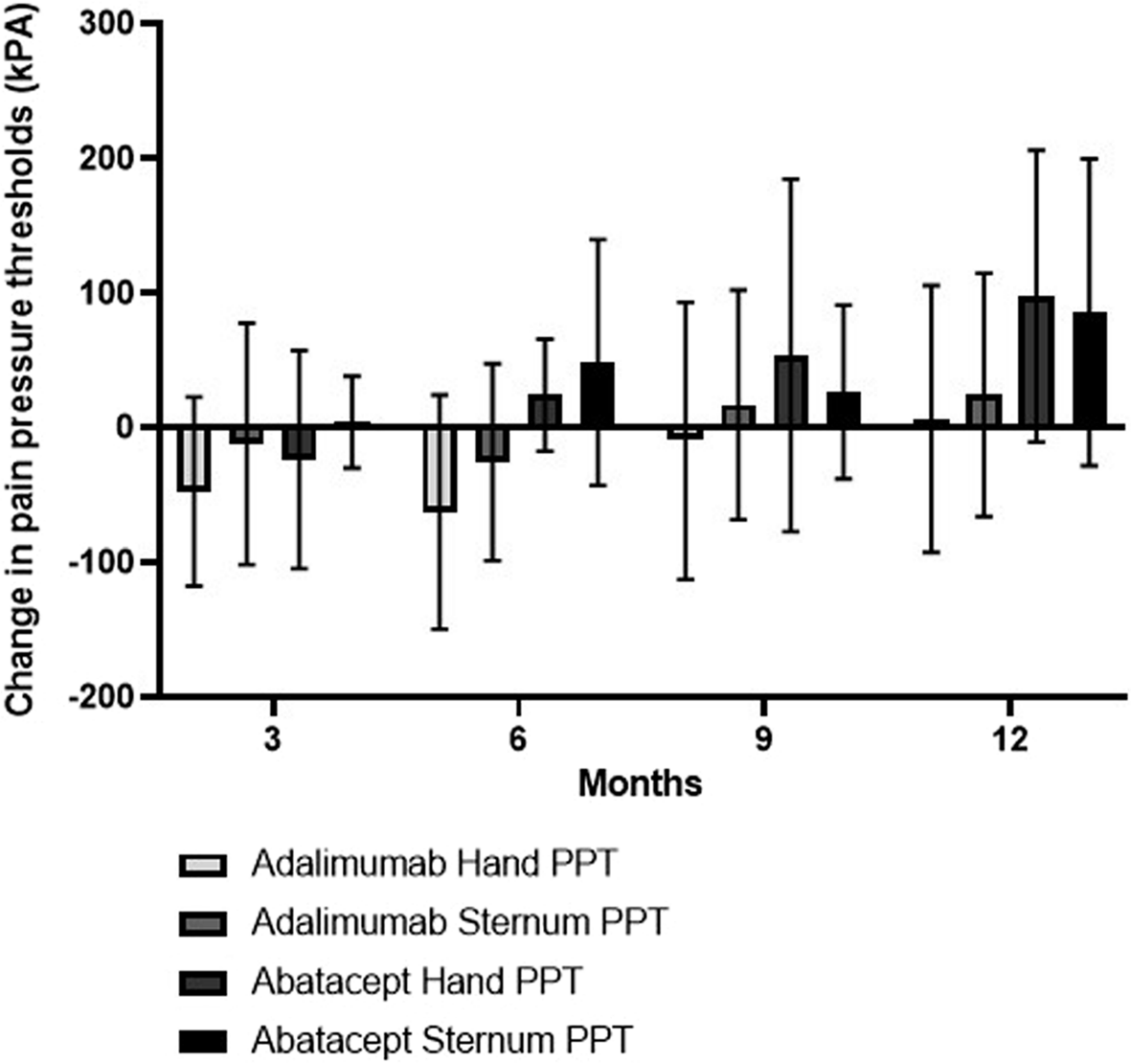
Graph illustrating change in pain pressure thresholds (PPT) measured by quantitative sensory testing (QST). Box and whisker plots show mean and standard deviations

There were two smokers in each of the adalimumab and abatacept groups. The mean BMI (SD) was 29.2 [12] in the abatacept group and 30.2 [7] in the adalimumab group. Pain outcome data is summarised in Figs. 2, 3, 4.

With respect to VAS pain, there was an improvement from baseline over 12 months of follow-up in VAS pain, with a mean of 3.7 (SEM 0.82) in the abatacept group and 2.3 (1.10) in the adalimumab group. For painDETECT measures, there was an improvement in mean painDETECT scores from baseline over 12 months of treatment 5.8 (SEM 1.93) in the abatacept group and 4.6 (2.54) in the adalimumab group. The change in PPT measures over 12 months is shown in Fig. 2. Higher (positive) values for PPT indicate a higher pain threshold, with subjects being able to tolerate more pain on pressure algometry. Our results show that the greatest improvement in PPT occurred in the abatacept group in the sternum (central sensitisation) and hand joints (peripheral sensitisation). More detailed data on PPT values is provided in Tables 2 and 3.

**Table 2:** Mean change from baseline and standard deviations for pain pressure thresholds (PPT) in the adalimumab and abatacept treatment groups

**Arm**	Months		*N*	Minimum	Maximum	Mean	Std. Deviation
**1** **Adalimumab**	3	Large Joint PPT	9	-253.42	65.83	-25.7	94.22
		Hand PPT	10	-162.69	35.34	-47.0	70.17
		Malleolus PPT	9	-376.67	37.33	-39.8	127.61
		Sternum PPT	9	-181.00	158.17	-11.5	89.58
	6	Large Joint PPT	9	-273.21	131.92	-15.3	117.17
		Hand PPT	9	-209.81	46.13	-62.3	86.64
		Malleolus PPT	9	-409.83	100.50	-13.7	153.54
		Sternum PPT	9	-182.83	66.17	-25.2	72.89
	9	Large Joint PPT	9	-217.29	203.21	2.6	113.91
		Hand PPT	9	-209.64	154.78	-9.3	102.80
		Malleolus PPT	9	-413.33	274.00	9.3	180.44
		Sternum PPT	9	-154.67	128.67	17.4	85.42
	12	Large Joint PPT	9	-229.33	145.79	9.9	116.26
		Hand PPT	9	-191.70	137.43	6.9	99.07
		Malleolus PPT	9	-415.67	214.67	7.5	172.88
		Sternum PPT	9	-164.83	124.67	24.7	90.50
**2** **Abatacept**	3	Large Joint PPT	9	-78.67	60.63	-5.9	51.35
		Hand PPT	9	-155.42	90.63	-23.3	80.68
		Malleolus PPT	9	-100.83	96.50	-14.5	74.55
		Sternum PPT	9	-31.67	77.67	4.6	33.98
	6	Large Joint PPT	7	-128.67	345.13	69.1	142.76
		Hand PPT	7	-34.77	69.57	24.5	41.63
		Malleolus PPT	7	-182.50	248.00	17.7	130.87
		Sternum PPT	7	-32.17	243.17	48.6	91.31
	9	Large Joint PPT	6	-135.21	181.08	37.3	122.29
		Hand PPT	6	-152.50	224.13	53.9	130.93
		Malleolus PPT	6	-160.50	138.33	16.7	111.80
		Sternum PPT	6	-48.50	114.17	26.7	64.61
	12	Large Joint PPT	4	-90.96	257.29	70.8	145.16
		Hand PPT	4	-13.57	243.70	97.9	108.27
		Malleolus PPT	4	-137.17	257.33	62.2	163.50
		Sternum PPT	4	-11.50	248.67	86.0	114.03

**Table 3:** Mean change from baseline and standard deviations for pain pressure thresholds (PPT) at the sternum in the adalimumab and abatacept treatment groups

**Arm**	CCP Status	Months	*N*	Minimum	Maximum	Mean	Std. Deviation
**1** **Adalimumab**	Negative	0					
		3	3	-16.00	33.67	0.7	28.58
		6	3	-3.00	26.50	10.9	14.82
		9	3	18.00	128.67	67.4	56.27
		12	3	55.33	119.17	79.1	34.89
	Positive	0					
		3	6	-181.00	158.17	-17.6	111.27
		6	6	-182.83	66.17	-43.3	85.06
		9	6	-154.67	70.33	-7.7	90.29
		12	6	-164.83	124.67	-2.4	99.79
**2** **Abatacept**	Negative	0					
		3	1	19.83	19.83	19.8	N/A
		6	1	53.83	53.83	53.8	N/A
		9	1	73.67	73.67	73.7	N/A
	Positive	0					
		3	8	-31.67	77.67	2.4	35.79
		6	6	-32.17	243.17	47.7	99.99
		9	5	-48.50	114.17	17.3	67.50
		12	4	-11.50	248.67	86.0	114.03

### Clinical characteristics

**Fig. 5:**
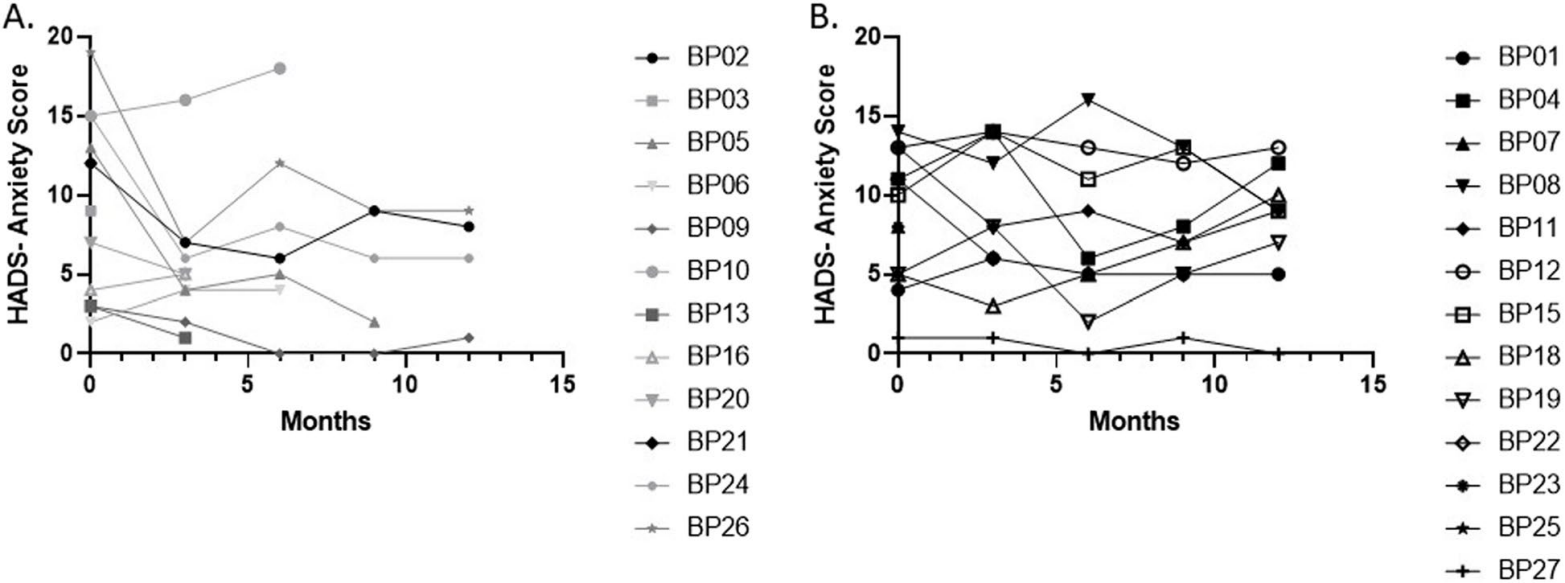
Participants HADS-Anxiety Scores over 12 months in the abatacept arm (**a**) and adalimumab arm (**b**) respectively

Participants were assessed for having anxiety or depression using the Hospital and Anxiety Depression scale (HADS). A score of 0–7 is normal, 8–10 borderline and above 11 abnormal. The mean HADS-Anxiety score in the abatacept group at the first visit was 9.5 (SEM 5.7), indicating on average borderline scores, and at 12 months it was 6 (SEM 3.6), indicating on average normal scores. The adalimumab group remained on average in the borderline region at 12 months, with a change from 8.8 (4.0) to 8.2 (3.9). This possibly indicates that there is an anxiolytic function associated with a greater reduction in pain and pain sensitisation with abatacept compared with adalimumab (Fig. 5).

In the HADS-Depression scores, both arms had a reduction in their mean scores. At baseline, the mean score in each group placed them in the normal or borderline range, with 7.5 (SEM 5.1) for abatacept and 8.5 (3.9) for adalimumab. At 12 months, they were 5.7 (3.8) and 6.0 (5.5) for adalimumab and abatacept respectively.

Both groups had a reduction in the mean HAQ scores at 12 months. Our results support real-world clinical evidence that biologics improve patients’ function. This correlates with the findings from the SF-36, which consists of 36 items organised into eight scales covering from physical function to emotional wellbeing. Specifically, the sub-section for pain showed an improvement in both groups, with the abatacept mean (SD) score 35.6 (18.61) increasing to 50.0 (21.79).

SF-36 and PROMIS-29 both have subsections related to pain (see Tables 4 and 5 for results). The mean PROMIS-29 pain inference score at baseline in adalimumab and abatacept was 63.5 (SEM 5.7) and 63.1 (4.8) respectively. Both groups showed an improvement in their pain inference as their scores reduced to 57.7 (4.4) for adalimumab and 57.7 (5.6) for abatacept. The other domains in these questionnaires are reported in the supplementary material.

**Table 4:** Mean change from baseline and standard deviations for SF36 pain score in the adalimumab and abatacept treatment groups

**Arm**	Months	*N*	Minimum	Maximum	Mean	Std. Deviation
**1** **Adalimumab**	0	13	0.00	0.00	0.0	0.00
	3	10	-45.00	35.00	1.3	21.99
	6	9	-45.00	55.00	1.11	30.70
	9	9	-32.50	45.00	7.8	20.82
	12	9	-55.00	45.00	8.6	32.48
**2** **Abatacept**	0	12	0.00	0.00	0.0	0.00
	3	9	-22.50	35.00	9.7	19.22
	6	7	0.00	37.50	15.0	11.90
	9	6	-5.00	77.50	33.8	26.68
	12	4	-5.00	22.50	10.6	11.43

**Table 5:** PROMIS-29 pain inference T scores in adalimumab and abatacept treatment groups

**Arm**	Months	*N*	Minimum	Maximum	Mean	Std. Deviation
**1** **Adalimumab**	0	13	55.60	75.60	63.5	5.71
	3	10	41.60	68.00	59.0	8.44
	6	9	52.00	69.70	59.2	6.48
	9	9	41.60	69.70	59.0	8.54
	12	9	49.60	63.80	57.7	4.36
**2** **Abatacept**	0	12	53.90	69.70	63.1	4.83
	3	9	53.90	66.60	59.5	4.42
	6	7	53.90	66.60	60.3	4.94
	9	6	41.60	65.20	51.6	11.18
	12	4	52.00	65.20	57.8	5.59

T-score explanation: PROMIS measures use a *T*-score metric in which 50 is the mean of a relevant reference population and 10 is the standard deviation (SD) of that population. On the T-score metric: A score of 40 is one SD lower than the mean of the reference population. A score of 60 is one SD higher than the mean of the reference population. For the PROMIS measures, a higher score equates to more of the concept being measured, e.g. more fatigue, more physical function. A score of 60 is one standard deviation above the average referenced population

Biomarkers such as ESR and CRP showed a reduction over the 12-month trial period; this is reflected in the disease activity score-28 which is used to monitor the disease severity. At 12 months, the DAS-28 score (SEM) had reduced by 2.5 (0.8) in the adalimumab group and 3.1 (1.9) in the abatacept group. This would meet the NICE criteria for a significant reduction to continue treatment.

### Adverse events

There were 83 recorded adverse events during the study, with 39 in the abatacept group and 44 in the adalimumab group. There was a total of 12 infections (6 due to COVID-19) in the abatacept group and 10 (three due to COVID-19) in the adalimumab group. There was one participant who reported flu-like symptoms after COVID-19 vaccination in the abatacept group and 3 in the adalimumab group. There was one transient ischaemic attack reported in the abatacept group which was not deemed to be related to the study drug. There was one SUSAR of bradycardia requiring hospital admission in the abatacept group, which led to withdrawal of the participant from the study. There was one death in the adalimumab arm from COVID-19 infection requiring hospitalisation. There were minor adverse events reported in the abatacept group (*n* = 19) and the adalimumab group (*n* = 18), including cough, itchy skin and injection site irritation.

## Discussion

Our study provides evidence for the first time that it is feasible to recruit, randomise and retain participants with active rheumatoid arthritis in a study assessing pain evaluation over 12 months. We found that assessing pain in people with RA is feasible and acceptable. During the study, we were able to conduct a comprehensive pain assessment for distinct modalities of pain in RA that addresses nociceptive, neuropathic and nociplastic pain elements over a sustained period of 12 months. Our study met its pre-defined progression criteria. We found that all participants remained in remission at the end of the trial and remained on biologic treatment after the study. Our exploratory analysis suggests that it is possible to stratify people for RA based on pain features. All the participants in our study had elements of inflammatory, nociplastic and neuropathic pain. Notably, all participants had high disease severity requiring biologic therapies.

Our experience of administering a large volume of questionnaires for pain, function and fatigue during the study highlighted that in future work, some of the questionnaires could be harmonised to use a smaller number of questionnaires which are sufficient to provide the information required for patient-reported clinical outcome measures. Although our study was not powered to detect a statistically significant difference between groups, we found that there was an improvement trend in VAS pain in the adalimumab and abatacept groups of by a mean of 2.3 and 3.7 points respectively. An improvement in neuropathic pain scores assessed by painDETECT was also observed in the two treatment groups, with an improvement of 4.6 in the adalimumab group and 5.8 in the abatacept group. There was an improvement in pain sensitisation measured by QST in both biologic groups, with a trend towards greater improvement in pain sensitisation measures in the abatacept versus the adalimumab group. The reason for a greater improvement in the hand and sternum regions may be due to abatacept targeting peripheral and central pain sensitisation in these areas in RA, but further work is required for validation.

Although a feasibility study, our data suggests that biologic therapies with different mechanisms of action may improve RA pain through distinct mechanisms. A greater improvement was observed in central (sternum) and peripheral (hand) pain sensitisation with abatacept compared with adalimumab after 12 months’ treatment. We found that use of abatacept, a T cell modulator, resulted in a greater improvement in pain sensitisation compared with adalimumab. Since T cells are involved in pain mediation, including by infiltrating nerves and impacting memory T cell function [23], further work is required to investigate the impact of T cell modulation on pain in RA.

Previous studies have shown that pain sensitisation [24], also described more recently as nociplastic pain [25], is an important component of pain perception in RA. It is described as an augmented pain and sensory perception with altered pain modulation and features of sensitisation that is often more widespread and intense than the level of tissue damage observed [25]. In one study, 139 subjects with RA were enrolled and underwent QST, including PPTs and temporal summation [26], RA disease activity was assessed using the Clinical Disease Activity Index (CDAI). The group found that low PPTs, indicating high pain sensitisation at all sites, were statistically associated with high CDAI scores (*p* < 0.03) and tender joints (*p* < 0.002). Measures of conditioned pain modulation (a measure of descending inhibitory pain pathways) were statistically associated only with tender joint count (*p* = 0.03). This study showed that high pain sensitisation was associated with high disease activity and other studies have also shown a similar effect. Another study showed that exercise had a positive influence on pain sensitisation in RA, where 53 subjects with chronic pain conditions, including 19 with chronic fatigue syndrome/fibromyalgia, 16 with RA and 18 healthy controls, found that exercise in people with RA improved temporal summation, thereby suggesting that several factors could modify pain responses in RA [27]. Recent work has also shown that newer biologic therapies, such as JAK (Janus kinase) inhibitors led to an improvement in pain sensitisation in people with central sensitisation and pain catastrophisation [28]

The observed reduction in pain on VAS, the proposed primary clinical outcome for a future confirmatory trial, was substantive and clinically meaningful. However, as a feasibility trial, we were reluctant to undertake a powered sample size calculation for a future trial based on the observed effects. The observed effects in feasibility trials are typically imprecise, whilst they may be further biased if the feasibility trial is not representative of any future trial [29]. In particular, the COVID pandemic affected recruitment and the sample size was much lower than originally planned. Therefore, the characteristics of the patients recruited and their observed treatment effects may have been unduly affected.

In people with RA, when inflammation is well-controlled but patients have ongoing pain, then fibromyalgia has been suggested as a mechanism for ongoing pain perception [30]. Features of fibromyalgia may be observed early in the disease when chronic pain activation pathways develop, and there is increasing recognition that fibromyalgia should be detected and treated early in the context of RA [30].

Limitations of our study were that we only tested 2 specific biologic agents targeted at TNF alpha and T cell modulation. It is possible that other DMARDs licensed for RA treatment could also have a beneficial effect on distinct pain modalities in RA. We also appreciate that since the study was conducted during the COVID-19 pandemic, this may have influenced the decision of some participants to get involved in research during the pandemic. We also conducted a qualitative sub study evaluating the experiences of our study participants during the COVID-19 pandemic [31]. Our participant interviews showed that some subjects were apprehensive about coming to the hospital for assessments and others felt isolated and ill-equipped to manage their symptoms. Future studies will need to factor in our observations from this study that some participants may be reluctant to engage in face-to-face studies due to concerns about COVID infection and effects of immunomodulation on their health.

In this feasibility study, we have demonstrated that it is practical to incorporate a comprehensive pain evaluation in a clinical setting for people with RA. Larger future studies are required to assess the relative importance of nociceptive, neuropathic and nociplastic elements during the natural history of RA which may assist in guiding future treatments. We have shown that QST is a practical tool that can be used clinically to assess central sensitisation with nociplastic pain features in RA.

## Data Availability

The trial essential documents and the trial database will be archived in accordance with the Sponsor (Joint Research and Enterprise Office, St George’s, University of London) SOP JREO SOP0016. The agreed archiving period for this trial will be 15 years. The data will be available for sharing by contacting the chief investigator.

## Abbreviations

BMI: Body mass index
CCP: Anti-cyclic citrullinated peptide antibodies
CDAI: Clinical Disease Activity Index
CONSORT: Consolidated Standards of Reporting Trials
CTLA-4: Cytotoxic T-lymphocyte associated antigen-4
CRP: C reactive protein
DAS-28: Disease Activity Score
DMARDs: Disease modifying anti-rheumatic drugs
ESR: Erythrocyte sedimentation rate
HADS: Hospital Anxiety and Depression Scale
HAQ-DI: Health Assessment Questionnaire-Disability Index
IMMPACT: Initiative on Methods, Measurement and Pain Assessment in Clinical Trials
JAK: Janus Kinase
MHRA: Medicines and Healthcare Products Regulatory Agency
MTX: Methotrexate
QST: Quantitative sensory testing
NICE: National Institute for Health and Care Excellence
PPT: Pain pressure thresholds
PROMS: Patient-related outcome measures
PROMIS-29: Patient Reported Outcome Information Reporting System
RA: Rheumatoid arthritis
TB: Tuberculosis
VAS: Visual analogue scale
